# Susceptibility to smoking and determinants among never-smoking high school students in Thailand

**DOI:** 10.18332/tid/156456

**Published:** 2023-01-11

**Authors:** Chakkraphan Phetphum, Atchara Prajongjeep, Waraporn Youngiam, Kanyarat Thawatchaijareonying

**Affiliations:** 1 Department of Community Health, Faculty of Public Health, Naresuan University, Phitsanulok, Thailand; 2 Tobacco Control Research Unit, Naresuan University, Phitsanulok, Thailand; 3 Department of Community Public Health, Sirindhorn College of Public Health Phitsanulok, Phitsanulok, Thailand

**Keywords:** smoking susceptibility, smoking, youth, cigarettes, never smoking

## Abstract

**INTRODUCTION:**

To prevent youth from becoming smokers, it is essential to understand factors contributing to them becoming susceptible to smoking. The aim of this study was thus to estimate the prevalence of smoking susceptibility among neversmoking youth in Thailand and to identify determinants associated with such behavior.

**METHODS:**

Cross-sectional data for 4572 eighth-grade students (aged 13–15 years) from 120 secondary schools were obtained from a classroom-based survey using a self-administered questionnaire. Using data from never-smoking students (n=3180), simple and multiple logistic regression analyses were performed to identify factors associated with smoking susceptibility. Frequencies and proportions for descriptive statistics are reported along with adjusted odds ratios and 95% confidence intervals for logistic regression models.

**RESULTS:**

A total of 16.4% of Thai never-smoking youth were susceptible to smoking. Several variables of interest were identified in multivariable analysis as significantly associated with increased susceptibility to smoking: being male (AOR=3.16; 95% CI: 25.4–3.92), having a positive attitude toward smoking – agreeing that smoking displays maturity (AOR=1.49; 95% CI: 1.07–2.09), the perception that smoking helps relieve stress (AOR=1.57; 95% CI: 1.14–2.15), the presence of current smoking peers (AOR=2.04; 95% CI: 1.57–2.66), exposure to secondhand smoking in public (AOR=1.51; 95% CI: 1.17–1.94), exposure to online cigarette and smoking-related advertising occasionally (AOR=1.98; 95% CI: 1.49–2.65), attendance at schools where there are sometimes anti-smoking education activities (AOR=1.57; 95% CI: 1.18–2.07); as well as exposure to anti-tobacco messages rarely (AOR=1.40; 95% CI: 1.05–1.87), occasionally (AOR=1.48; 95% CI: 1.12–1.96) and infrequently (AOR=1.41; 95% CI : 1.07–1.87).

**CONCLUSIONS:**

Approximately one in six Thai never-smoking youth was found to be susceptible to smoking. Findings suggest it should be useful to supplement relevant tobacco prevention and control efforts by considering such interpersonal and socio-environmental determinants, among vulnerable people to smoke.

## INTRODUCTION

As a leading cause of preventable diseases, cigarette smoking is responsible for 6 million deaths worldwide every year^1^, with 90% of smokers starting during their teenage years^2^. Despite a decline in smoking among global youth aged 13–15 years^3^, the number of youths using alternative smoking products is likely to remain stable or will tend to increase^4^. Additionally, 11.9% of smoking naïve adolescents across the world were susceptible to smoking, which varied from 8.6% in South-East Asia to 17.9% in the Americas (categorized by WHO regions)^4^.

Additionally, 12.5% of young people (7.2% male, 5.3% female) worldwide who never smoked, were susceptible to smoking^4^. Never-smoking youth showed varying degrees of susceptibility to smoking across the six WHO regions, from 10.1% in South-East Asia to 29.8% in Europe^4^.

Among never-smoking youth in high-income countries, 16.5% were susceptible to tobacco use, compared to 11.5% in low- and middle-income countries^5^. Therefore, in addition to tobacco control strategies and effective prevention programs for smokers, youth who have never smoked should also be prevented from becoming susceptible and developing the habit.

Smoking susceptibility refers to current non-smokers who are predisposed or motivated to start a smoking trial in the future^6^. According to the stage-of-change model, those who are susceptible to smoking begin by preparing for smoking by thinking about smoking, forming opinions, and developing beliefs about tobacco use, and this can eventually lead to the initiation, experimentation, regular smoking, and addiction^4,7^. Susceptibility to smoking can be developed during childhood^8^ and is a highly predictive variable leading to smoking initiation^8-10^, and current use of combustible cigarette^9^ as well as alternative modern tobacco products such as electronic cigarettes and hookahs^8-11^. Identifying non-smoking youth who are vulnerable to smoke is, therefore, a prerequisite for preventing smoking initiation among young people^12^.

A range of intrapersonal and socio-environmental determinants has been found to be associated with smoking susceptibility among non-smoker youth. The intrapersonal factors include being male^4,12^, having positive attitudes toward smoking^13-15^, having a low level of knowledge regarding the harm of smoking^12,14,15^, and having a low perception of the smoking threats^16-18^. In terms of socio-environmental factors, peer smoking^13,15,19^, secondhand smoke exposure indoors and outdoors^5,12,14^, inadequate parental supervision against smoking^14^, attending at a school without anti-smoking activities^4^, not being exposed to anti-smoking messages^4,12^, and noticing tobacco advertising and promotion^4,14^ have been indicated to contribute to smoking susceptibility. Although these variables have been studied extensively, they have been found to be relatively limited among youth in the Thai context.

Youth smoking prevalence has been a public health concern in Thailand. According to the Global Youth Tobacco Survey (GYTS), school-based surveys of high school students aged 13–15 years, in 2005, 2009, and 2015, have shown that the smoking prevalence of Thai youths has been consistently high at approximately 11%^20^. All forms of tobacco use were three times more common in boys than in girls^21^. Nearly half of children and youth have been exposed to secondhand smoke, with 47.9% exposed in public and 46.8% at home^21^. Among youth who have never smoked, 7.4% were likely to smoke or use alternative tobacco products in the future^20^.

Currently, there is limited evidence on susceptibility to smoking among Thai youth aged 13–15 years. This research aims thus to examine the prevalence and determinants related to smoking susceptibility among these younger youth. This study was a cross-sectional national survey collecting data from youth representatives from all regions of Thailand. The findings of this study will be useful as a reference at the population level and for developing effective preventive interventions for reducing young non-smokers’ susceptibility to smoking, which should limit the likelihood of future early-stage smoking trials.

## METHODS

### Study design, setting and participants

This study involved a cross-sectional survey conducted between May and August 2019, and was a school-based study collecting data from a total of 120 public and private secondary schools, located in 12 provinces covering all regions of Thailand. The sample consisted of eighth-grade school students aged 13–15 years. Those who did not obtain parental/guardian consent or refused to participate in the survey were excluded. The students who finally participated were asked to complete a questionnaire voluntarily and anonymously, and a project researcher was present to assist and address any concerns as needed. Confidentiality was ensured by using anonymous questionnaires and no exposure of the questionnaire to others. The study was approved by the Ethics Committee in Human Research at Naresuan University, and was not funded by tobacco-related organizations or companies.

### Sampling method

The sample size was estimated by using the infinite population proportion, with p=0.074 (based on a previous national survey in Thailand which found that 7.4% of non-smoking youth were likely to smoke cigarettes or use alternative tobacco products in the future)^20^, and Delta=0.0111 [15% of p; α=0.05; Z(0.975)=1.96; and design effect=2]. To account for non-responses or incomplete responses, a 10% was added to the calculated sample number. Therefore, the final sample size was 4572 people.

Sampling was conducted using a stratified two-stage cluster sampling scheme. First, a stratified random sampling approach was used to select four regions of Thailand: the north, center, northeast, and south. Second, three representative provinces from each region with a total of 12 provinces were selected using a cluster random sampling method (a lottery method). Third, a cluster random sampling (a lottery method) was applied to draw ten schools from each selected province with a total of 120 schools. Lastly, another cluster random sampling method (a lottery method) was used to select an eighth-grade classroom from each school. In total, 4655 students, who exceeded the calculated sample size, were randomly selected, and invited to the survey. Based on the data integrity check, the response rate was 85.54%.

### Measures

The self-administered questionnaire (in the Thai language) was developed based on previous studies^6,22^ and the researchers. The questionnaire was examined for Integrity of Content (IOC) by three experts in the fields of behavioral science and youth tobacco control, before pilot testing it with 30 eighth-grade students not included in the study sample. No incidence reports were received by the researchers during the data collection period.

Only non-smokers, those students who responded ‘Not at all’ to ‘Have you ever smoked in your lifetime?’, were included in the analysis. The dependent variable was susceptibility to smoking, measured by applying reliability and a valid series of questions developed by Pierce et al.^6,22^ including: 1) ‘If one of your best friends were to offer you a cigarette, would you smoke it?’; 2) ‘Do you think you will be smoking cigarettes 5 years from now?’; and 3) ‘At any time in the next 12 months do you think you will smoke a cigarette?’. Four response options were given for these questions: ‘definitely not’, ‘probably not’, ‘probably yes’ and ‘definitely yes’. Respondents who answered, ‘definitely not’ to all questions were considered unsusceptible to smoking and recorded as ‘0’, while those who answered other combinations were classified as susceptible to smoking and recorded as ‘1’^10,11,23-25^. This score has an internal reliability of 0.7218 (Cronbach’s α) and has proven to be a valid predictor of future smoking initiation^6^.

Following existing literature, a total of 16 independent variables that could contribute to smoking susceptibility in never-smoking youth was investigated. The first ten items were used to assess intrapersonal and socio-environmental determinants: 1) gender (male/female); 2) age (13, 14 and 15 years); 3) current smoking parents (yes/no); 4) smoking peers (yes/no); 5) exposure to secondhand smoke at home during the past 30 days (yes/no); 6) exposure to secondhand smoke in public during the past 30 days (yes/no); 7) exposure to online tobacco advertising (often, sometimes, never); 8) parental supervision against smoking (often, sometimes, never); 9) anti-smoking education at school (often, sometimes, never); and 10) exposure to anti-tobacco messages (often, sometimes, never). Another three items with three response options (disagree, unsure, agree) were used to measure perceived health risks associated with smoking: 11) ‘Young smokers are at a lower risk of health hazards from smoking than adults’; 12) ‘Young people who smoke never or occasionally have a lower risk of becoming addicted to cigarettes’; and 13) ‘Menthol cigarettes are less harmful than unflavored cigarettes’. A final three items with three response options (disagree, unsure, agree) were used to assess attitudes toward smoking: 14) ‘Youth who smoke display maturity’; 15) ‘Smoking helps reduce stress’; and 16) ‘Young smokers are more attractive to the opposite sex’.

### Statistical analysis

We performed using SPSS version 17.0 for Windows (SPSS Inc., Chicago, Illinois). Sociodemographic characteristics among never-smoking high school students were described by frequency and percentage. Chi-squared was calculated to compare incorrect perceptions and inappropriate attitudes between the susceptible to smoking group and the unsusceptible to smoking group. Simple logistic regression was used to analyze the crude relationship between the key factors of interest as independent variables and susceptibility to smoking. To identify predictors of smoking susceptibility, a multivariable logistic regression model was used with the Wald backward method, to control the effect of the relationships among the independent variables until the adjusted odds ratio (AOR) was obtained. Results are presented as AOR compared to crude odds ratios (OR), with a 95% confidence interval (CI).

## RESULTS

Following the exclusion of 802 students who smoked cigarettes during their lifetime, the remaining sample of this study was 3180 never-smoking youth. Among this group, 405 students reported they would smoke a cigarette if their friends offered them one, 448 students reported they will be smoking in five years, and 357 students reported they will smoke next year. In total, the eligible samples who were susceptible to smoking were 521 (16.4%).

**Figure 1:**
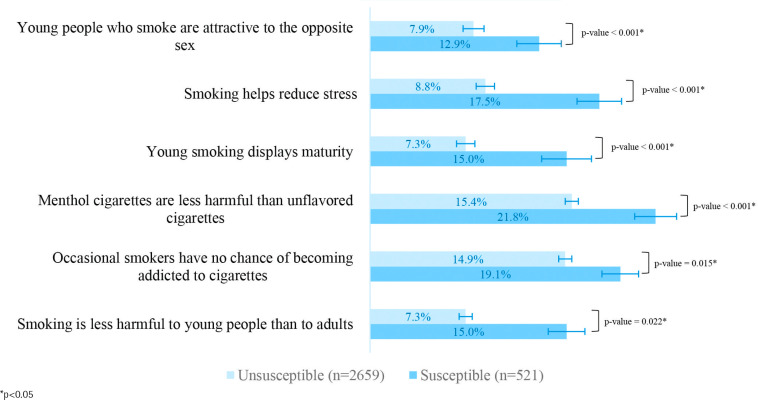
The proportions of inaccurate risk perceptions and positive attitudes toward smoking between smoking susceptible and unsusceptible students, Thailand, 2019 (N=3180)

The majority of the susceptible students were males (70.1%) and aged 14 years (58.3%). The most common exposure among this group was never or sometimes noticing anti-tobacco messages (58.2%), followed by spotting online cigarette advertising frequently (49.5%), and attending schools where anti-smoking education events were sometimes or never held (39.9%). On all questionnaires regarding perceived health risks and attitudes towards smoking, the susceptible group was found to have a higher proportion of incorrect perceptions and negative attitudes than the unsusceptible group (Figure 1).

The bivariate analyses of factors associated with smoking susceptibility among never smoking youth found that all variables, except age, current parental smoking and exposure to secondhand smoke at home, were correlated with smoking susceptibility at the 0.05 significance level (Table 1).

**Table 1:** Sociodemographic characteristics among never-smoking high school students and crosstab analysis classified by smoking susceptibility, Thailand, 2019 (N=3180)

*Characteristics*	*Total (N=3180) n (%)*	*Susceptible (N=521) n (%)*	*Unsusceptible (N=2659) n (%)*	*Crude odds ratio*		
				*OR*	*95% CI*	*p*
**Gender** (n=3157)						
Female (Ref.)	1679 (53.2)	148 (8.8)	1531 (91.2)	1		
Male	1478 (46.8)	368 (24.9)	1110 (75.1)	3.43	2.79–4.21	<0.001*
**Age** (years) (n=3153)						
13 (Ref.)	997 (31.6)	162 (16.2)	835 (83.8)	1		
14	1878 (56.6)	304 (16.2)	1574 (83.8)	0.99	0.83–1.19	0.918
15	278 (8.8)	52 (18.7)	226 (81.3)	1.14	0.84–1.54	0.395
**Current parental smoking** (n=3180)						
No (Ref.)	2423 (76.2)	387 (16.0)	2036 (84.0)	1		
Yes	757 (23.8)	134 (17.7)	623 (82.3)	1.14	0.95–1.36	0.154
**Current peer smoking** (n=3179)						
No	2789 (87.7)	397 (14.2)	2392 (85.8)	1		
Yes	390 (12.3)	124 (31.8)	266 (68.2)	2.81	2.21–3.56	<0.001*
**Exposure to secondhand smoke at home** (n=3180)						
No (Ref.)	2423 (76.2)	387 (16.0)	2036 (84.0)	1		
Yes	757 (23.8)	134 (17.7)	623 (82.3)	1.17	0.97–1.42	0.107
**Exposure to secondhand smoke in public** (n=3155)						
No (Ref.)	2356 (74.7)	338 (14.3)	2018 (85.7)	1		
Yes	799 (25.3)	180 (22.5)	619 (77.5)	1.74	1.42–2.13	<0.001*
**Exposure to online cigarette advertisement** (n=3160)						
Never (Ref.)	843 (26.7)	158 (18.7)	685 (81.3)	1		
Sometimes	396 (12.5)	102 (25.8)	294 (74.2)	2.24	1.72–2.90	<0.001*
Often	1921 (60.8)	258 (13.4)	1663 (86.6)	1.49	1.20–1.85	<0.001*
**Parental supervision against smoking** (n=3164)						
Often (Ref.)	2455 (77.6)	373 (15.2)	2082 (84.8)	1		
Sometimes	320 (10.1)	58 (18.1)	262 (81.9)	1.66	1.28–2.15	<0.001*
Never	389 (12.3)	89 (22.9)	300 (77.1)	1.24	0.91–1.68	0.174
**Anti-smoking education at school** (n=3156)						
Often (Ref.)	2285 (72.4)	310 (13.6)	1975 (86.4)	1		
Sometimes	422 (13.4)	91 (21.6)	331 (78.4)	2.25	1.76–2.86	<0.001*
Never	449 (14.2)	117 (26.1)	332 (73.9)	1.75	1.35–2.27	<0.001*
**Exposure to anti-tobacco messages** (n=3155)						
Often (Ref.)	1709 (54.2)	215 (12.6)	1494 (87.4)	1		
Sometimes	604 (19.1)	117 (19.4)	487 (80.6)	1.97	1.59–2.45	<0.001*
Never	842 (26.7)	186 (22.1)	656 (77.9)	1.67	1.30–2.14	<0.001*
**Smoking is less harmful to young people than to adults** (n=3170)						
Disagree (Ref.)	2651 (83.6)	385 (14.5)	2266 (85.5)	1		
Unsure	247 (7.8)	58 (23.5)	189 (76.5)	1.51	1.11–2.05	0.008*
Agree	272 (8.6)	78 (28.7)	194 (71.3)	1.15	0.90–1.47	0.253
**Occasional smokers have no chance of becoming addicted to cigarettes** (n=3157)						
Disagree (Ref.)	2234 (70.8)	340 (15.2)	1894 (84.8)	1		
Unsure	432 (13.7)	79 (18.3)	353 (81.7)	1.25	0.95–1.63	0.109
Agree	491 (15.6)	99 (20.2)	392 (79.8)	1.41	1.08–1.80	0.007*
**Menthol cigarettes are less harmful than unflavored cigarettes** (n=3157)						
Disagree (Ref.)	2179 (69.0)	317 (14.5)	1862 (85.5)	1		
Unsure	459 (14.5)	88 (19.2)	371 (80.8)	1.39	1.07–1.81	0.013*
Agree	519 (16.4)	113 (21.8)	406 (78.2)	1.64	1.29–2.08	<0.001*
**Young people who smoke are attractive to the opposite sex** (n=3141)						
Disagree (Ref.)	2653 (84.5)	391 (14.7)	2262 (85.3)	1		
Unsure	215 (6.8)	61 (28.4)	154 (71.6)	2.29	1.67–3.14	<0.001*
Agree	273 (8.7)	67 (24.5)	206 (75.5)	1.88	1.40–2.53	<0.001*
**Young smoking displays maturity** (n=3170)						
Disagree (Ref.)	2651 (83.6)	385 (14.5)	2266 (85.5)	1		
Unsure	247 (7.8)	58 (23.5)	189 (76.5)	1.81	1.32–2.47	<0.001*
Agree	272 (8.6)	78 (28.7)	194 (71.3)	2.37	1.78–3.14	<0.001*
**Smoking helps reduce stress** (n=3171)						
Disagree (Ref.)	2562 (80.8)	369 (14.4)	2193 (85.6)	1		
Unsure	284 (9.0)	60 (21.1)	224 (78.9)	1.59	1.17–2.16	0.003*
Agree	325 (10.2)	91 (28.0)	234 (72.0)	2.31	1.77–3.02	<0.001*

* p<0.05.

With regard to the multivariable logistic regression analysis, males who had never smoked a cigarette were significantly more susceptible to smoking than females (AOR=3.42; 95% CI: 2.83–4.12). Regarding smoking attitudes, never-smoking youths who were unsure (AOR=1.43; 95% CI: 1.09–1.39) and agreed (AOR=1.80; 95% CI: 1.39–2.31) that smoking helps reduce stress were significantly more likely to be susceptible to smoking. Never smokers who had smoking peers were most associated with increased smoking susceptibility (AOR=2.04; 95% CI: 1.62–2.57), followed by those who sometimes noticed online cigarette advertising (AOR=1.98; 95% CI: 1.49–2.64), and those exposed to secondhand smoke in public (AOR=1.51; 95% CI: 1.17–1.94). Furthermore, young non-smokers who attended schools where anti-smoking education activities were provided sometimes (AOR=1.34; 95% CI: 1.05–1.71) or never (AOR=1.42; 95% CI: 1.11–1.82), were statistically significantly more susceptible to smoking than students who attended schools with a higher frequency of anti-smoking education activities. Additionally, young non-smokers exposed to anti-tobacco messages occasionally (AOR=1.53; 95% CI: 1.21–1.94) or never (AOR=1.31; 95% CI: 1.03–1.67), were significantly more likely to smoke than those exposed consistently (Table 2).

**Table 2:** Crude odds ratio and adjusted odds ratio of factors associated with susceptibility to smoking among never-smoking high school students, Thailand, 2019 (N=3180)

*Factors*	*OR*	*95% CI*	*p*	*AOR^a^*	*95% CI*	*p*
Male gender (Ref.: female)	3.43	2.79–4.21	<0.001*	3.42	2.83–4.12	<0.001*
Current peer smoking (Ref.: no)	2.81	2.21–3.56	<0.001*	2.04	1.62–2.57	<0.001*
Exposure to secondhand smoke in public (Ref.: no)	1.74	1.42–2.13	<0.001*	1.47	1.18–1.82	<0.001*
**Exposure to online cigarette or smoking advertising** (Ref.: never)						
Sometimes	2.24	1.72–2.90	<0.001*	1.98	1.49–2.64	<0.001*
**Anti-smoking education at school** (Ref.: often)						
Sometimes	2.25	1.76–2.86	<0.001*	1.34	1.05–1.71	0.020*
Never	1.75	1.35–2.27	<0.001*	1.42	1.11–1.82	0.005*
**Exposure to anti-tobacco messages** (Ref.: often)						
Sometimes	1.97	1.59–2.45	<0.001*	1.53	1.21–1.94	<0.001*
Never	1.67	1.30–2.14	<0.001*	1.31	1.03–1.67	0.027*
**Smoking helps reduce stress** (Ref.: disagree)						
Unsure	1.59	1.17–2.16	0.003*	1.43	1.09–1.39	0.011*
Agree	2.31	1.77–3.02	<0.001*	1.80	1.39–2.31	<0.001*

a AOR: adjusted odds ratio; adjusted for all variables listed. Hosmer-Lemeshow test step 7. χ^2^=10.63; df=7; p =0.156.

* p<0.05.

## DISCUSSION

Youth smoking has been a major public health threat in Thailand and the prevalence of young smokers is unlikely to decline^20^. Approximately one-tenth of Thai youths currently smoke^21^. The current circumstances are likely to persist, as the present study revealed that nearly one in six Thai never-smoking youth were susceptible to smoking. The smoking susceptibility prevalence (521/3180; 16.4%) found in this study is higher than that of the Thai survey of 2015 (7.4%), the average among South-East Asian youth survey (10.1%), and the average of Global Youth Survey (12.5%)^4^, while it is similar to that in Malaysia (14%)^12^. In contrast, this study’s prevalence was lower than that of Europe (29.8%) and the Americas (26.5%)^4^. In part, this relatively high prevalence of smoking susceptibility among Thai youths may have contributed to the high rates of smoking in Thailand. The reasons for this could be that young people who smoke are partially motivated and live in a society and an environment that is conducive to smoking.

Susceptibility to smoking is the first experience in life that leads people to hesitate or think about starting smoking at the pre-experimental stage^8-11^, and susceptible people could then develop more advanced stages of smoking^9^. According to various evidence, adolescents who are not current smokers but are susceptible to smoking are 2–3 times more likely to use cigarettes in the future than youth who are not susceptible^4-11^. Therefore, in addition to implementing strong tobacco control strategies and an effective prevention campaign targeting smokers, it is crucial to prevent never-smoking youth from becoming susceptible and developing a smoking habit.

According to the present findings, most intrapersonal and socio-environmental factors of interest are likely to be factors for the susceptibility to smoking among young non-smokers. As in previous studies^4,12^, male youth were more likely to smoke than female youth. Because smoking susceptibility is the first experience that leads to youth smoking, the fact that the prevalence of susceptibility among males is higher than females is plausible and consistent with the smoking situation among Thai youths, where males smoke at a rate higher than females in all previous surveys^20^.

Based on previous studies^13,14,16^, young people who did not currently smoke but had a positive attitude towards smoking were more likely to be more susceptible to smoking than those with a more negative attitude. In this study, youth who agreed that smoking helps reduce stress were approximately 1.8 times more likely to be susceptible to smoking than those who disagreed. These findings reflect the need for interventions for correcting inappropriate attitudes among young people.

Consistent with previous studies^13,15,19^, this study found that approximately one-tenth (12.3%) of the current non-smoking students had at least one close friend who smoked, and these students were twice as susceptible to smoking than those without a smoking peer. These circumstance can be explained by the fact that current smoking-free students who share their daily school life with smokers, regard smoking as socially acceptable. The non-smoking youth thus have an increased possibility of accepting cigarettes from their peers, thereby increasing the likelihood that they will attempt to smoke for the first time^4,15^.

Exposure to secondhand smoke is another environmental risk factor for youth smoking. Similar to previous studies^4,12,14^, the results of this study found that exposure to secondhand smoke outdoors increased youth susceptibility to smoking by 1.5 times, compared to smoke-free environments in public. As in a previous study^14^, this study found no relationship between secondhand smoke exposure in the students’ homes and smoking susceptibility. Perhaps smoking by parents is a behavior that students have been accustomed to since childhood, and so this is not a novel experience compared to smoking by people of the same age. Therefore, the legislation relevant to smoke-free public places should be enforced more rigorously.

In this study, students who were often or sometimes exposed to cigarette advertising on the internet were nearly twice as likely to be susceptible to smoking as students who were never exposed to such commercials, in line with previous findings^4^. This may be the result of online tobacco advertising providing students with the ease of accessing and purchasing cigarettes online without barriers associated with age verification. Online smoking scenes may also positively influence perceptions that smoking as normal and be an incentive for future smokers to be more willing to try their first cigarette. Despite Thailand’s efforts to ban cigarette advertising since 1992, online channels are not effectively controlled. Therefore, government agencies should develop proactive counter-technology measures by increasing the level of surveillance, investigation, and prosecution of violators.

As far as school anti-tobacco campaigns are concerned, the results of this study are similar to those of a previous study^4^ that determined that never smokers who received irregular anti-smoking campaigns at school were approximately 1.5 times more likely to become smokers than students attending schools with regular activities. According to these findings, anti-smoking health education strategies should be implemented in schools, which is consistent with a Malaysian study indicating that non-smokers who were educated about the dangers of smoking reduced their chances of ever smoking in the future^12^.

Consistent with the previous research^4,12^, this study also found that non-smoking students who never encountered anti-smoking messages were nearly 1.5 times more likely to be susceptible to smoking than those who saw such messages often. Daily exposure to tobacco risks and anti-smoking messages may also deflect a tobacco company’s marketing strategy geared toward young customers, which might otherwise have negative effects on students’ attitudes and behaviors toward smoking^4^. Therefore, it is vital to design anti-smoking messages and communicate them to youth consistently and appropriately without interference from tobacco companies. Additionally, a successful anti-smoking advertising campaign designed by young people for young people should be considered to prevent the risk factors and promote the preventive factors.

### Strengths and limitations

This study has several strengths. First, this study was one of a few to investigate whether youth who had never smoked cigarettes were susceptible to smoking. Such studies were limited in middle- and low-income countries, including in Thailand^26^. Second, this current study examined a range of the determinants of susceptibility to smoking, including personal, psychological and environmental factors. Third, the data were collected from a large sample group and covered all regions of Thailand; therefore, it can be generalized to a larger population. Lastly, it adopted the internationally validated measure of smoking susceptibility that can be compared with other countries and monitored in the WHO database. However, this study has a number of limitations. First, it relied exclusively on self-reports, which were likely to be impacted by reporting bias. Smoking or the intention to smoke may not be socially acceptable behavior. Consequently, there could also be a social desirability bias in the report. Second, although this cross-sectional study reports associations between the variables, their causality cannot be determined. In spite of that, this study used a large sample to infer causality, and its findings met several criteria, including the reliability and consistency of some associations, as well as the plausible nature of their effects. Future studies will require longitudinal surveillance data to examine smoking susceptibility and initiation. Third, generalizing the results of this study should be taken with caution. Considering that this survey was conducted among adolescents enrolled in secondary schools, it may not be representative of the entire young population. Fourth, the analysis of this study did not control for other substance use such as alcohol or illicit drugs, which have been also indicated to be associated with smoking. Finally, this measure of smoking susceptibility among never smokers uses the original three questions developed by Pierce et al.^6,22^ and not an updated version that includes a question about ‘curiosity’ that assesses both traditional and alternative tobacco products^1,15^. By using this measure, the rate of smoking initiation can be better predicted and adolescents who are vulnerable to smoking can be identified more accurately^1,15^. Despite the limitations, the current study offers valuable insight into the prevalence of smoking susceptibility and its factors among Thai youths.

## CONCLUSIONS

Smoking susceptibility among those who never smoked is a significant predictor of future smokers. It is thus imperative to limit the incidence of new smokers by preventing non-smokers, especially young people, from becoming susceptible to such behavior. The findings indicate that a considerable number of youths in Thailand are susceptible to smoking. Several interpersonal and socio-environmental determinants including male gender, positive smoking attitudes, peer smoking, exposure to secondhand smoke in public, exposure to online cigarette advertising, less exposure to school anti-smoking education, and less exposure to anti-tobacco messages, were identified as being associated with increased smoking susceptibility among the vulnerable population. To diminish the likelihood of becoming susceptible to smoking and future smokers among young people, it would be vital to complement prevention and control efforts, following the WHO-FCTC, concerning such determinants targeting this young population. Additionally, the Ministry of Public Health and Education should integrate and implement programs or campaigns at the individual, school, and community levels. As young people constitute a valuable population in society, maintaining youth healthy lifestyles is crucial for the population’s well-being.

## Data Availability

The data supporting this research cannot be made available for privacy or other reasons.
